# Aquaporins as diagnostic and therapeutic targets in cancer: How far we are?

**DOI:** 10.1186/s12967-015-0439-7

**Published:** 2015-03-21

**Authors:** Jian Wang, Li Feng, Zhitu Zhu, Minghuan Zheng, Diane Wang, Zhihong Chen, Hongzhi Sun

**Affiliations:** Department of Pulmonary Medicine, Zhongshan Hospital, Fudan University, Shanghai, China; Minghang Hospital, Fudan University, Shanghai, China; The First Hospital of Liaoning Medical University, Jingzhou, China

**Keywords:** AQPs, Cancer, Water channel, Inhibition, Biomarkers

## Abstract

Aquaporins (AQPs) are a family of water channel proteins distributed in various human tissues, responsible for the transport of small solutes such as glycerol, even gas and ions. The expression of AQPs has been found in more than 20 human cancer types and is significantly correlated with the severity of histological tumors and prognosis of patients with cancer. More recent evidence showed that AQPs could also play a role in tumor-associated edema, tumor cell proliferation and migration, and tumor angiogenesis in solid and hematological tumors. Inhibitors of AQPs in tumor cells and microvessels have been suggested as new therapeutic strategies. The present review overviews AQPs structures, expression variation among normal tissues and tumors, AQPs functions and roles in the development of cancer with special focuses on lung, colorectal, liver, brain and breast cancers, and potential AQPs-target inhibitors. We call the special attention to consider AQPs important as diagnostic and therapeutic biomarkers. It may be a novel anticancer therapy by the AQPs inhibition.

## Introduction

The aquaporins (AQPs) are a family of small, transmembrane, water-transport proteins distributed in various human tissues [1]. Thirteen members in AQPs family have been identified in humans, divided into two groups according to transported materials. For example, AQP1, AQP2, AQP4, AQP5, or AQP8 are exclusively selective for water, while AQP3, AQP7, AQP9, or AQP10 proposed as aquaglyceroporins can transport water and small neutral solutes such as glycerol [2]. Recently AQPs such as AQP1, AQP4, or AQP5 were found to be permeable for ion and gas flow (e.g. O_2_, CO_2_, or nitric oxide) [3-5]. Most AQPs are located in plasma membrane to drive osmotic-gradients-dependent water transport, while AQP11 and AQP12, as the super-aquaporins, are expressed in cytoplasm to regulate intracellular water transport, organelle volume, or intra-vesicular homeostasis [6]. AQP4 has multiple isoforms, although the number of AQP4 isoforms remains uncertain [7,8]. AQP4-M1 isoform is the full-length protein with the sequence starting at methionine 1, whereas AQP4-M23 isoform starts at methionine 23 without the first 22 amino acid sequences [9]. AQP4-M1 and AQP4-M23 can form supra-molecular structures as orthogonal arrays of particles (OAPs), of which the size is positively associated with M23:M1 ratio [10]. OAPs assemblies were suggested to be required for NMO-IgG to recognize AQP4 in neuromyelitis optica (NMO), although the function of OAPs was still unclear [11].

A large number of studies showed that AQPs were closely associated with cancer biological functions and expressed in more than 20 human cancer cell types [9]. AQPs expression is positively correlated with tumor types, grades, proliferation, migration, angiogenesis, or tumor-associated edema [2,12,13], which can be considered as diagnostic and therapeutic targets in anticancer treatment. For example, AQP1 and AQP4 were massively up-regulated in high-grade astrocytomas, as compared with low-grade ones and normal brain tissues [13,14]. The high expression of AQP4 was correlated with the formation of more prominent brain edema [15]. AQP1 was up-regulated in lung adenocarcinoma and inhibition of AQP1 expression can inhibit tumor cell invasion, which thereby were proposed as the prognostic index and therapeutic target for lung cancer [16]. Fortunately, many AQPs-target inhibitors have been developed to damage tumor cells. But further researches were required to verify the efficiency and safety of these AQPs-target inhibitors in clinical therapy. The present review overviews AQPs structures, AQPs expression in normal and tumor tissues, AQPs functions and specific roles in cancer development, and potential AQPs-target anticancer drugs.

### AQPs structures

**Figure 1:**
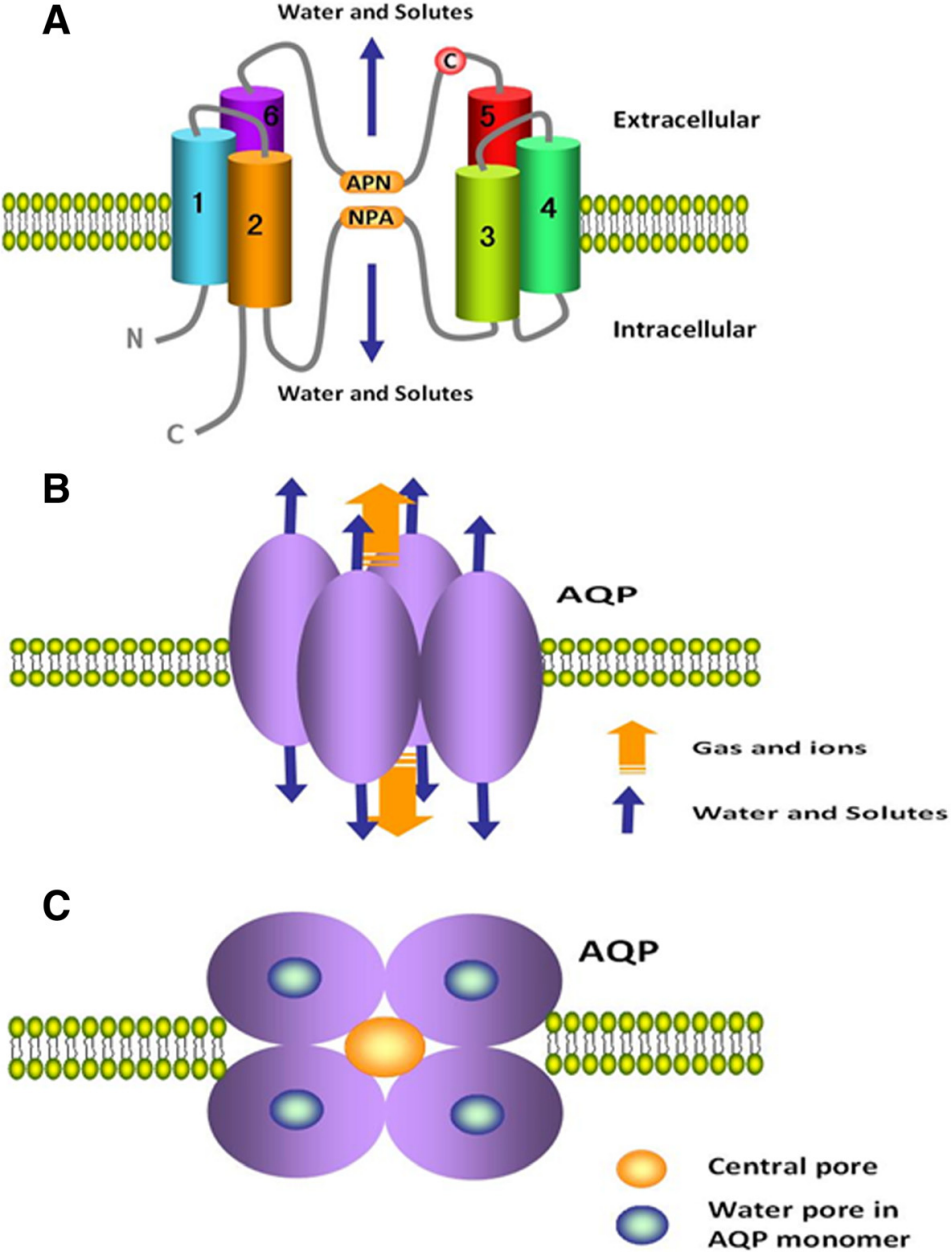
**The structures of AQP monomer and homotetramers. (A)** Each AQP monomer has six titled α-helical domains to form a water pore spanning plasma membrane. Conserved sequence motifs NPA on the loops bend into molecule to pair with each other and form the water channel. C, in red, represents a cysteine residue (Cys 189) that can block the AQPs function with functional sensitivity to mercury. **(B)** The structure of AQP homotetramers from side view. Each AQP monomer contains independently a water pore. AQP monomers assemble as homotetramers to form a central pore in homotetramers. Red arrow represents the central pore with transporting gas and ions. Blue arrow represents the water pore with transporting water and solutes. **(C)** The structure of AQP homotetramers from the top view. Each AQP monomer contains independently a water pore. AQP monomers assemble as homotetramers to form a central pore in homotetramers.

Each AQP monomer weighs about between 28 and 30 kDa and has six-titled α-helical domains to form a barrel-like structure spanning plasma membrane (Figure 1A). The polypeptide in the structure is formed by a single chain with about 270 amino acids, and amino (N) and carboxyl (C) terminals are located in the cytoplasm [17]. Two highly conserved sequence motifs asparagine-proline-alanine (NPA) with a short helix are located on opposite sides of monomer. NPA motifs bend into molecule to pair with each other and form the water channel [18]. A cysteine residue (Cys 189) can be found in most members of AQPs family and resides near the channel in extracellular orientation, which can block AQPs with functional sensitivity to mercury [19].

AQP monomer contains independently water pore and assembles as homotetramers to contribute to fluid transport [20] (Figure 1B). As for the narrow pore with the diameter of about 2.8 Å at the narrowest point and electrostatic interactions, AQP1, AQP2, AQP4, AQP5, and AQP8 mediate water translocation in single file and block proton transport [17,21,22]. This process is dependent on osmotic gradients without ATP expense [17]. As to aquaglyceroporins, AQP3, AQP7, AQP9, and AQP10 contain two additional peptide spans and modulate the size of pore with a diameter of 3.4 Å at the narrowest point) to transport glycerol [20,23].

AQP monomers assembly forms a central pore in homotetramers (Figure 1C). Several studies indicated that the central pore was permeable for gases O_2_, CO_2_, or nitric oxide in AQP1, AQP4, and AQP5 [3-5]. The AQPs-dependent gas transport is faster than free diffusion and plays an important role in the biological function. For example, AQP1-dependent CO_2_ transport in proximal tubules of the kidney regulates arterial pH during metabolic acidosis, possibly by acting as a CO_2_ transporter [24,25]. The ability of aquaporins to transport NO may tightly control NO concentrations in target cells and directional releases from NO-producing cells mediates NO-dependent relaxation in the vasculature [26,27]. However, the molecular mechanism of gas transport through the central pore remains unclear.

### AQPs expression in normal and cancer tissues

Human AQPs distribute in different normal tissue and most of them are located in epithelium and endothelium, as well as some other typical cells such as erythrocytes, astrocytes, adipocytes and skeletal muscle [28]. AQP1, AQP3, AQP8 and AQP9 have a wide expression in a variety of human tissues and act as special functions in different organs [17]. AQP0 is expressed in human lens, involving in lens transparency and homeostasis [29]. AQP2 and AQP6 are mainly found in kidney and regulate the urinary concentration [30,31]. The significant role of AQP4 is involved with brain edema for its up-expression in stroke, trauma, tumor and other central nervous system (CNS) disorders, although it is normally located in perivascular astrocyte foot processes [32]. AQP5 is mainly expressed in lung, gastrointestinal tract and secretory glands [33], while AQP7 is found in adipocytes, skeletal muscle, heart and kidney [17].

**Figure 2:**
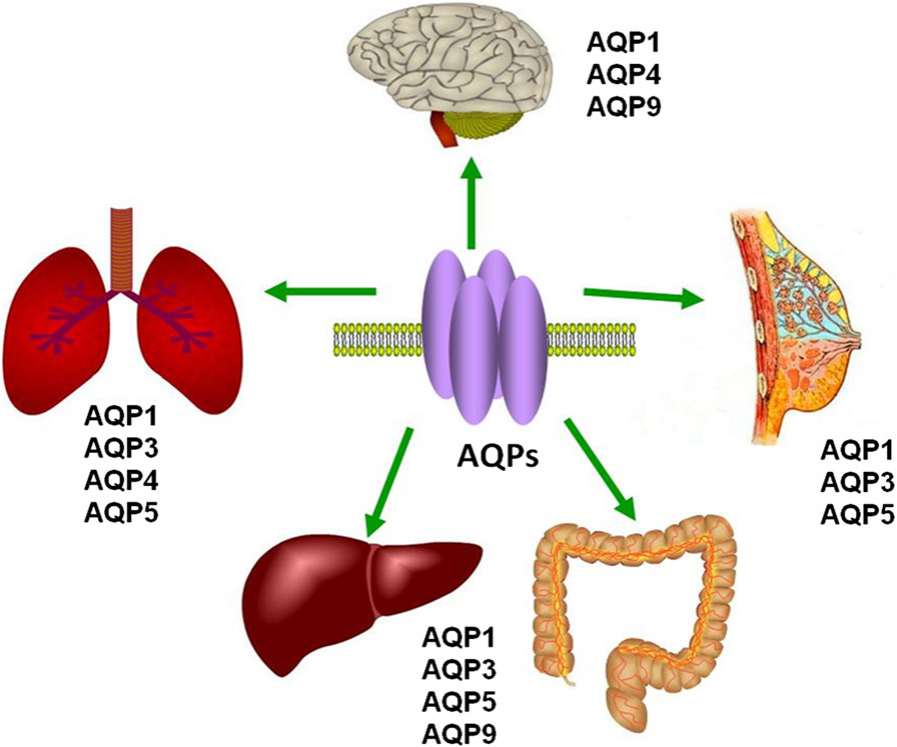
**The over-expression of AQPs in cancers as elaborated in the review.** The role of AQPs in lung, colorectal, liver, brain and breast cancers has been widely studied.

AQPs have been found to express in several solid and hematological cancer cells. The detail of AQPs expression in different cancer tissues is summarized in Table 1. One type of AQP protein can be expressed in different cancer tissues and there is a lack of the tumor-specific prosperity. AQP1 is over-expressed in brain, lung, breast, colorectal, or ovarian cancers [13,34-37]. The expression of AQP3 is up-regulated in cutaneous, esophageal and oral squamous, pulmonary, renal, or hepatocellular cancers [38-42]. The expression of AQP4 is increased in brain, lung, or thyroid cancers [14,43,44]. AQP5 is over-expressed in lung, chronic myelogenous leukemia (CML), ovarian, stomach, or colorectal cancers [45-49]. AQP7 expression is increased in thyroid cancer, while AQP9 mainly in brain or ovarian cancers [50-52]. However AQP8 is down-expressed in hepatocellular or colorectal cancers [53,54]. In other hands, one type of cancer can also present a close association with several different AQPs. The AQPs in lung, colorectal, liver, brain and breast cancers are showed in Figure 2.

**Table 1:** **AQPs expression in different human tumors**

**Aquaporins**	**Tumor type**	**Correlated function**
AQP1	Brain cancer [13], Breast cancer [35], Colorectal cancer [34], Cervical cancer, Laryngeal cancer, Lung cancer [43], Nasopharyngeal cancer, Ovarian cancer [37]	Tumor grade, prognosis, tumor angiogenesis, tumor necrosis, tumor cell migration, tumor invasion and metastasis.
AQP3	Colorectal cancer [34], Cervical cancer, Liver cancer [40], Lung cancer [9], Oesophageal cancer [39], Renal cancer [41], Skin cancer [38], Stomach cancer, Tongue cancer [42]	Tumor grade, prognosis, tumor angiogenesis, tumor invasion, tumor cell migration and tumor energy metabolism.
AQP4	Brain cancer [14], Lung cancer [43], Thyroid cancer [44]	Tumor grade, migration, tumor-associated edema, adhesion, invasion and tumor apoptosis.
AQP5	Breast cancer [9], Colorectal cancer [34,45], Cervical cancer, Leukaemia [46], Liver cancer [40], Lung cancer [47], Oesophageal cancer [39], Ovarian cancer [48], Stomach cancer [49], Tongue cancer [42]	Tumor prognosis, tumor proliferation, tumor invasion, tumor cell migration and drug resistance.
AQP7	Thyroid cancer [50]	Unknown
AQP8	Cervical cancer	Tumor cell migration, tumor invasion, tumor metastasis and anti-apoptosis.
AQP9	Brain cancer [51], Ovarian cancer [52]	Tumor grade, drug resistance and energy metabolism.

### AQPs functions

The physiological and pathological functions of AQP proteins have been investigated in the condition of AQP deletion. In normal human tissues, AQPs regulate brain water homeostasis, exocrine gland secretion, urine concentration, skin moisturization, fat metabolism or neural signal transduction. In the CNS, AQP4 plays a principle role in the regulation of blood-brain barrier (BBB) water permeability while AQP1 facilitates cerebrospinal fluid secretion. The deletion AQP4 can reduce water permeability through the cell plasma membrane in the brain [55-57]. In the salivary glands, AQP5 transports osmotic water into acinar lumen, and regulates the production, viscosity, or tonicity of the saliva [58-60]. Several AQPs (AQP1, AQP2, AQP3, or AQP4) are important in regulating the balance of water in different locations of kidney. The deletion of AQP1 or loss of function mutations in AQP1 could compromise the ability to concentrate the urine maximally during the challenge with water deprivation [61,62]. AQP2 is regulated by vasopressin (AVP) and AQP2 deletion leads to severe polyuria with a low urine osmolality [63]. In normal skin, the basal layer keratinocytes of epidermis express AQP3, involving in water and glycerol transport and skin hydration [64,65]. Deletion of AQP3 reduces skin elasticity and skin hydration, reduces biosynthesis of the stratum corneum, or delays wound healing [66]. Besides, AQP7 is suggested to regulate fat metabolism of adipocytes, due to the increased body fat mass, defective glycerol exit, and consequent accumulation of glycerol and triglycerides during AQP7 deletion [67]. The role of AQPs in neural signal transduction is unclear, but AQP4 expression on astroglia is proposed to regulate the volume of the extracellular space, interact with Kir4.1 K^+^ channels, or balance the water re-uptake into astroglia during neural signaling [1].

**Figure 3:**
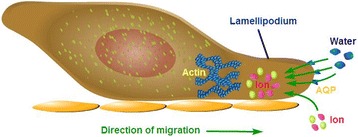
**The proposed mechanism of AQP-dependent cell migration.** Actin depolymerization and ion movement at the tip of a lamellipodium increase local osmolality that drives water influx to form membrane protrusion.

AQPs also facilitate cell proliferation, migration, and adhesion or angiogenesis in the tissue, which may play an important role in the pathogenesis of cancer. It was reported that vascular endothelial cells expressed AQP1 to play a key role in the pathogenesis of tumor angiogenesis by accelerating cells migration [68]. AQP1-null mice had low ability to form the angiogenesis in the cancer tissue induced by subcutaneous injection of melanoma cells and produced extensive tumor necrosis. Cancer cells with AQP1 over-expression had stronger capacity of cell migration, invasion, and metastasis [69]. AQP1 is thus proposed as a key player in cancer biology and a potential target for drug development. Likewise, AQP4 was strongly expressed in brain and facilitates astroglial cell migration and scarring formation after brain injury, which could be prevented by the deletion of AQP4 [70,71]. It was proposed that the water influx at the tip of a lamellipodium through actin cleavage and ion uptake resulted in the membrane protrusion responsible for the AQP-dependent migration [1] (Figure 3). AQP4 was also considered as a critical control factor in cell adhesion in the ocular lens [72,73].

### AQPs in lung cancer

AQP1, AQP3, AQP4 and AQP5 are over-expressed in lung cancer [74,75]. AQP1 was mainly up-expressed in lung adenocarcinoma (ADC) and bronchoalveolar carcinoma (BAC) instead of lung squamous cell carcinoma and normal lung tissue [16]. The over-expressed AQP1 was located in the endothelial cells of capillaries within lung cancer tissue responsible for the development of angiogenesis [43,76]. AQP1 could regulate lung cancer cell invasion and migration which can be inhibited by AQP1-shRNA [43]. The expression of AQP1 was correlated with high postoperative metastasis ratios and low disease-free survival rates in ADCs, especially with micropapillary ADC components [75]. It was proposed that AQP1 could be a significant prognostic index for stage and histologic differentiation of lung cancer.

AQP3 was over-expressed in non-small cell carcinoma (NSCLC), especially adenocarcinomas, or well-differentiated bronchioloalveolar carcinomas and papillary subtypes. It is possible that AQP3 may regulate biological functions of lung cancer cells and be required in the early stage of lung ADC development [77]. AQP3 may be involved in the initiative of angiogenesis in lung cancer through HIF- 2α-VEGF pathway, lung cancer cell invasion partly by the AKT-MMPs pathway, cellular glycerol uptake, or mitochondrial ATP formation [78]. The anticancer effects of shRNA-targeting AQP3 were observed in experimental NSCLC, further evidenced by the inhibition of AQP3 deletion for lung cancer growth and prolonged survival in preclinnical studies [78].

AQP5 over-expression was observed predominantly in lung ADCs and associated with poor prognosis of patients with NSCLC. AQP5-positive cells exhibited a loss of epithelial cell markers and activation of c-Src by AQP5 through SH3 binding domain to promote EMT, which might contribute to the enhanced metastasis potential of lung cancer [47]. Over-expressed AQP5 could facilitate lung cancer cell growth and invasion through the activation of the EGFR/ERK/p38 MAPK pathway [79]. The cAMP-protein kinase (PKA) consensus site in AQP5 was preferentially phosphorylated and promoted cell proliferation ability in tumor. The phosphorylation at Ser156 in PKA consensus site was demonstrated as a key role in tumor proliferation and invasion by Ser156 mutants in lung cancer cells [80]. Thus, Ser156 in AQP5 provided a unique opportunity as therapeutic target. We can design small molecule inhibitors to inhibit the phosphorylation of Ser156 or its downstream pathway as a new anticancer therapy. Developing AQP5-specific monoclonal antibody will also be another approach we can take in the future.

### AQPs in colorectal cancer

AQP1, AQP3, AQP5 and AQP9 are found in colorectal cancer. AQP1 had the oncogenic property in colon cancer and was over-expressed in the early stage of the disease and through the late stage of colorectal carcinogenesis [34]. AQP1 expression was correlated with the progression of colon cancer, including lymph node metastasis and lymphovascular invasion. AQP1 was thus proposed as an independent poor prognostic factor in colon cancer [81]. Forced-expressed AQP1 in colon cancer cells increased the plasma membrane water permeability and migration ability, which could be inhibited by AQP1-specific blockers. The mechanism of AQP1-induced cells migration and metastasis in colon cancer cells was the relocalization of actin protein and activation of RhoA and Rac [82]. So downregulation expression of AQP1 in colorectal cancer may be a new therapeutic approach.

The colon cancer had higher expression of AQP3 compared to the normal tissue. The expression intensity of AQP3 was correlated with tumor differentiation and metastasis in colon cancer patients. The migration of colon cancer cells can be inhibited by AQP3-specific inhibitor, as well as EGFR pathway inhibitors [83]. It provided a new therapeutic strategy in colon cancer by blocking AQP3 and EGFR pathway.

AQP5 was over-expressed in colorectal cancer and a strong prognostic biomarker for colorectal cancer. A positive relationship between AQP5 over-expression and the number of circulating tumor cells was observed in colon cancer [84]. Patients with colon cancer expressed with AQP5 had more chances to appear liver metastasis. The over-expression of AQP5 promoted cell proliferation, while deletion of AQP5 inhibited colon cancer cell growth. AQP5 induced tumor proliferation by the activation of Ras-MAPK pathway, cyclin D1/CDK4 complexes, and then phosphorylated retinoblastoma protein in nucleus and caused transcription of genes related with cell proliferation [45].

Besides, AQP5 and AQP9 were associated with the resistance of colon cancer to drugs. Deletion of AQP5 could increase sensitivity to chemotherapeutic drugs by the inhibition of p38 MAPK pathway in colon cancer cells [85]. Down-expression of AQP9 was related with non-responses of colorectal cancer cells to adjuvant chemotherapy [86]. Thus, AQP5-p38 MAPK pathway and AQP9 would be a new therapeutic target to improve drug resistance of colon cancer.

### AQPs in hepatic cancer

Hepatic cancer expresses similar types of AQPs with colon cancer, e.g. AQP1, AQP3, AQP5, and AQP9. AQP1 expression was specially found in cholangiocarcinomas (CCs) and the microvessels of hepatocellular carcinomas (HCCs) while was negative in HCCs and metastatic colorectal carcinomas (MCCs) [87]. The immunohistochemical detection of AQP1 can be more reliable to differentiate CCs from HCCs and MCCs, especially in tumors of which histology alone is not sufficient for a proper diagnosis. AQP1 is thus suggested as the potential diagnostic factor to differentiate diagnosis in liver cancer pathologies.

AQP3 and AQP5 were over-expressed in HCCs and negatively associated with 5-year disease-free survival and 5-year overall survival. It was suggested that the combination of AQP3 and AQP5 protein expression could be an independent poor prognostic factor for patients with HCCs. Besides, co-expression of AQP3 and AQP5 in HCCs has a significant association with serum AFP and tumor stage and grade. It may be helpful to diagnose HCCs by combining serum AFP and AQP3 and AQP5 [40].

The expression of AQP9 was down-regulated in HCCs and mainly located in non-tumourigenic liver tissue [88]. Deceased AQP9 expression in HCCs can increase resistance of hepatic cancer cells to apoptotic stimulation [53]. It indicated that AQP9 would be a new diagnostic or therapeutic target in hepatic cell cancer.

### AQPs in brain cancer

**Figure 4:**
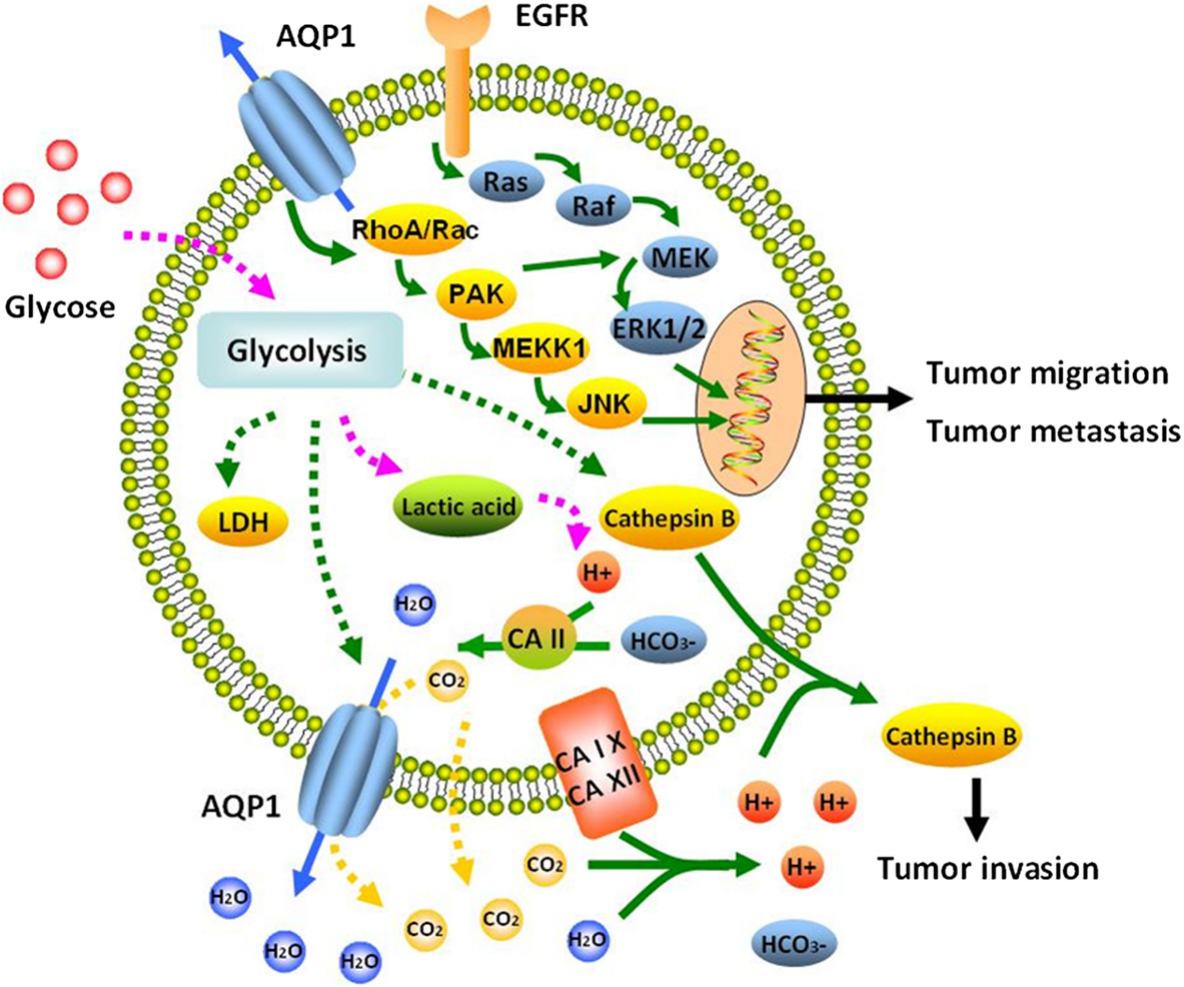
**Proposed model of novel role of AQP1 in tumor biology.** Tumor cells increase glycose consumption to produce lactic acid, which results in excess H^+^ production and intracellular acidosis. The increase in glycolytic intermediates may up-regulate AQP1, LDH, and cathepsin B through the E-box/ChoRE. Excess H^+^ and HCO_3_^-^ are catalyzed by intracellular CAII to produce H_2_O and CO_2_. The reaction-generated H_2_O is transported to extracellular space to aviod cytotoxic edema by up-regluated AQP1. CO_2_ may or may not leave the cells through AQP1. Membrane-bound extracellular CA IX and XII may regenerate H^+^ from extracellular H_2_O and CO_2_, thus leading to shutting H^+^ from the intracellular to the extracellular space to keep the acidification of the extracellular compartment. The acid extracellular environment promotes cells to release cathepsin B, a proteolytic enzyme involved in tumor invasion. AQP1 can induce the activity of RhoA and Rac to increase tumor migration and metastasis. The detailed role of AQP1 in this pathway need more studies.

Astrocytic glioma is the most common primary brain cancer in humans, of which glioblastoma multiforme as grade IV glioma is the most malignant primary brain tumor with high mortality and poor prognosis [89]. AQP1, AQP4, and AQP9 are over-expressed in brain tumors and proposed to play an important role in carcinogenesis. AQP1, expressed normally in the choroid plexus epithelium, was over-expressed and up-regulated in all types of glioblastoma and associated with the severity and grades of astrocytoma [13,90,91]. The over-expression of AQP1 was predominantly located in perivascular areas or areas of tumor cell infiltration, distant from the necrotic tumour core [92]. It suggested that AQP1 expression was strongly correlated with tumor angiogenesis and proposed as a therapeutic target in brain cancer. Glioma cells present high rates of aerobic glycolysis, resulting in increased lactic acid production. AQP1 can combine with carbonic anhydrases to shut H^+^ from the intracellular to the extracellular compartment, leading to prevention from tumor cytotoxic edema and acidification of extracellular compartment. The acid extracellular environment promoted glioma cells to release cathepsin B, which increased the invasion of glioma cells [93] (Figure 4).

AQP4 expression was up-regulated and redistributed in gliomablast accompanied by a loss of its polarized expression pattern, and played a role in the brain edema formation [15]. AQP4 over-expression in human astrocytoma was associated with the presence of brain edema detected by the magnetic resonance imaging [2]. The gilomablast was characterized with peritumoral edema and considered as vasogenic brain edema [94]. The redistribution of AQP4 in glioblastoma cells was explained as a reaction to VEGF-induced vasogenic edema in order to facilitate the reabsorption of excessive fluid [95]. It remained to be clarified whether increased expression of AQP4 enhanced edema formation or clearance. AQP4 deletion reduced glioma cell migration and impaired the polymerization of F-actin which is involved in cell-cell adhesion and movement [96]. It was suggested that AQP4 was involved in the control of glioblastoma cell migration and invasion through cytoskeleton rearrangement and cell adhesion regulation [97]. AQP4 was also proposed as an anti-apoptosis target for therapy of glioblastom, evidenced by the finding that siRNA-mediated down-regulation of AQP4 induced glioblastoma cell apoptosis [98].

The over-expression of AQP9 was observed across the whole surface of human glioma cells, associated with the energy metabolism, and counteracted the glioma-associated lactic acidosis by clearance of glycerol and lactate from the extracellular space [99]. The over-expression of AQP9 in astrocytic glioma was associated with the pathological grades [51], indicating that AQP9 can be a diagnostic biomarker and therapeutic target for brain cancer therapy.

### AQPs in breast cancer

The expression of AQP1, AQP3, and AQP5 is up-regulated in breast cancer. AQP1 was mainly over-expressed in the cytoplasm of breast cancer cells and correlated with poor prognosis of patients with breast cancer as an independent prognostic factor [100,101]. The potential mechanism by which AQP1 was involved in breast tumor growth and metastasis may be that AQP1 induced the development of angiogenesis by stimulating and activating endothelial cells through estrogen receptors in human breast cancer. Estrogen can also induce AQP1 expression by activating estrogen-response element (ERE) in the promoter of the AQP1 gene, resulting in tubulogenesis of vascular endothelial cells [102].

AQP3 was found as a critical and necessary factor for the migration of human breast cancer cells induced by fibroblast growth factor-2 (FGF-2) [103]. FGF-2 could increase AQP3 expression and cell migration through FGFR-PI3K or FGFR-ERK signaling pathways, which was blocked by the deletion of AQP3. AQP3 expression in breast cancer cells was increased by the stimulation with 5′-deoxy-5-fluoropyrimidine nucleosides (5′-DFUR), which was used in the chemotherapy of solid tumors [104]. It was proposed that AQP3 may be a limiting factor in pharmacological effects of 5′-DFUR, since the deletion of AQP3 reduced the efficacy of the drug. It seemed that AQP3 can be considered as a chemotherapeutic target to develop the combining strategy for cancer treatment. However, it should be questioned whether AQP3 can act as a breast cancer-specific diagnostic biomarker or therapeutic target.

AQP5 was mainly expressed at apical domains of ductal epithelial cells in breast benign tumor, while such apical polarity of AQP5 in ducts was lost in invasive ductal carcinoma [105]. AQP5 was suggested to regulate the proliferation and migration of breast cancer cells and indicate the prognosis for the patients, probably associated with the estrogen receptor/progesterone receptor or epidermal growth factor receptor 2 [106]. AQP5 over-expression was proposed as an independent prognostic marker of survival for breast cancer patients with estrogen-positive tumor.

### Therapeutic targets

AQPs has been identified in different cancers and associated with tumor proliferation, metastasis, angiogenesis, tumor cell adhesion and tumor-associated edema. Thus, it will be an attractive way for us to make AQPs as the therapeutic targets in anticancer treatment. Now, AQPs-target inhibitors including cysteine-reactive heavy metal-based inhibitors, small-molecule inhibitors which inhibit AQPs expression or AQP-induced water permeation, and monoclonal AQP-specific antibody, have been developed and verified. It is also inspiring that the AQP gene transfer has been developed and applied to clinical therapy that eleven participants with previously irradiated parotid glands after radiation therapy for head or neck cancer received AQP1-cDNA transfer therapy in the Phase I clinical trial [107]. However, the efficacy and safety of AQP gene transfer need more studies.

As mentioned above, mercury can block the water-transport function in AQPs with Cys 189, such as AQP1. Similarly, heavy metal-based AQPs inhibitors such as silver-, gold- or ruthenium-containing compounds were considered as potential anticancer drugs [108,109]. For example, the common chemotherapy drug cisplatin inhibited the expression of AQP5 which is up-expressed in ovarian tumor and associated with lymph node metastasis and ascites [110]. There are also several small-molecule compounds without heavy mental, which can block AQPs to inhibit tumor biological functions. Acetazolamide, carbonic anhydrase inhibitor, was found to inhibit the expression of AQP1 which protected tumor from cytotoxic edema by maintenance of extracellular acidification and promoted tumor metastasis in glioma [111]. Besides, several AQP1-target inhibitors including cyclophosphamide, topiramate and anesthetic drugs [112], AQP4-target inhibitors including anti-epileptic drugs, bumetanide, sumatriptan and thiadiazole [9], and AQPs-target inhibitors including TEA^+^ [113] were reported in succession. However, the following studies failed to verify AQPs inhibition of these small-molecule AQPs-target inhibitors by different assays [113].

The AQP4-IgG detected in patients with NMO is specially bound to AQP4 and inhibits AQP4 water permeability, thereby leading to complement-dependent cytotoxicity in astrocytes [114]. Now, the monoclonal AQP4-specific antibody has been developed [115]. Thus, the speculation that AQP4-specific antibody linked with toxin can be used to selectively damage AQP4-expressing glioblastoma cells has been proposed [9]. It is still full of surprise and trouble in the way for developing AQPs-target inhibitors as potential anticancer drugs.

### Conclusion and perspective

**Figure 5:**
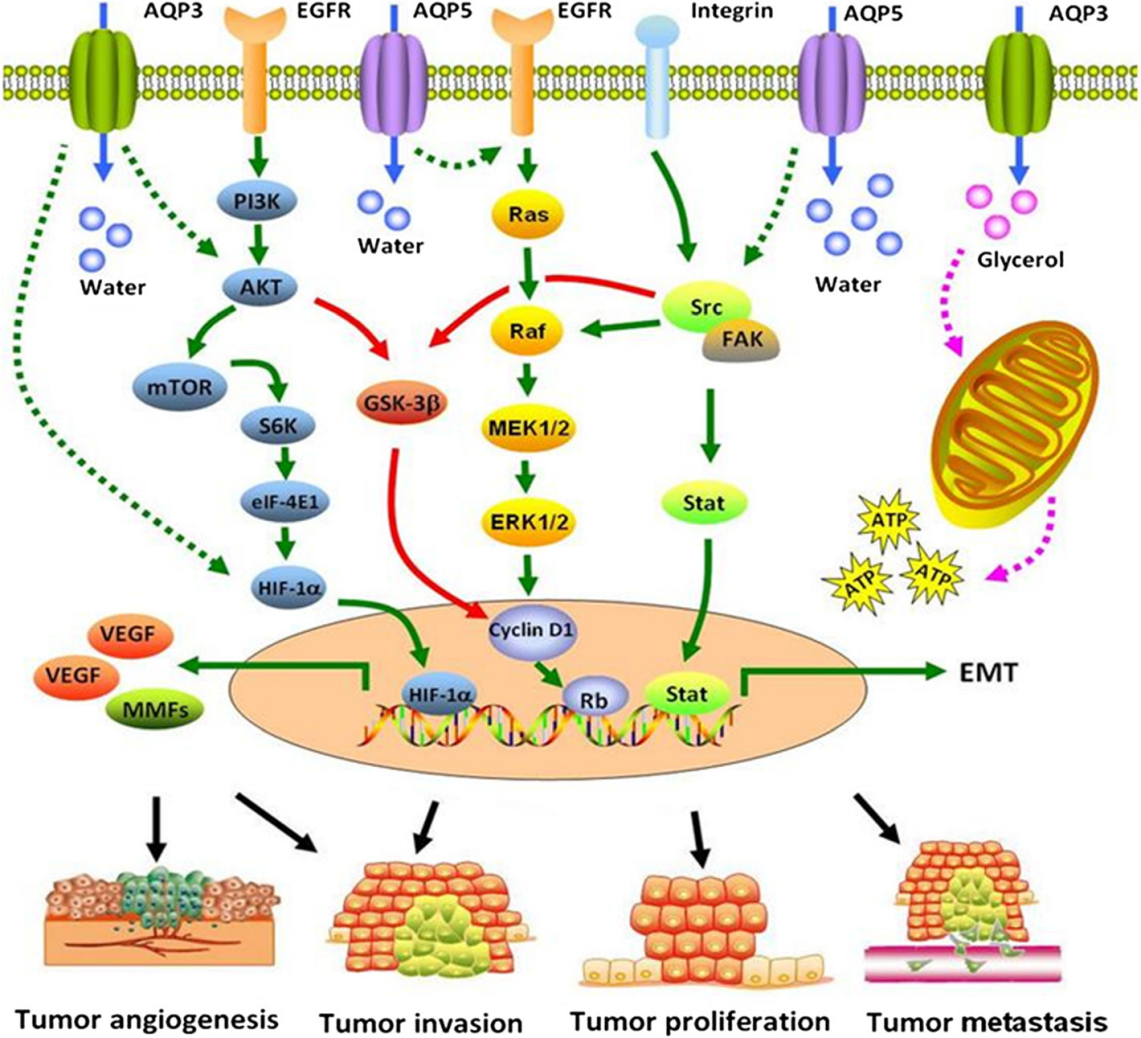
**Proposed model of novel roles of AQP3 and AQP5 in tumor biology.** AQP5 are exclusively selective for water while AQP3 can transport water and other small neutral solutes such as glycerol. AQP3 increases intracellular glycerol content which is transported to mitochondria to form ATP. Mitochondrial ATP formation provides energy for tumor cell proliferation. AQP3 can directly or indirectly reduce the natural degradation of HIF-1α/ HIF-2α to increase the expression of vascular endothelial growth factor (VEGF), which is a critical regulator in tumor angiogenesis and vessel maturation. AQP3 may directly or indirectly activate AKT to increase MMPs, resulting in tumor invasion. AQP5 induces activation of the epidermal growth factor receptor (EGFR), extracellular receptor kinase (ERK1/2) pathway to faciliate tumor cell proliferation and metastasis. In addition, AQP5 is phosphorylated on Ser156 and bind the the SH3 domain of Src to promote EMT activity in tumor cells. The roles of AQP3 and AQP5 in these pathways are not fully elucidated and still need further exploration.

Clinical and preclinical studies evidence that the expression of AQPs increase in a number of cancers. Biological functions and singling pathways of AQPs in cancer are intensively investigated in condition of AQP deletion (Figure 5). AQPs play an important role in brain water homeostasis, exocrine gland secretion, urine concentration, skin moisturization, fat metabolism or neural signal transduction. AQPs are also involved in the carcinogenesis and pathogenesis of tumor-associated edema, tumor cell proliferation and migration, or tumor angiogenesis. The results from studies in vivo or vitro also show the attractive opportunities for AQPs-target therapy. AQPs-target inhibitors and AQPs-specific monoclonal antibody, even AQP gene transfer, are developed to provide new therapeutic strategy in anticancer treatment. However, there is still a long way to clearly elucidate the specificity of AQPs in the pathogenesis, metabolisms, and controls of various cancers, or resistance and tolerance to anticancer therapies. The process in AQPs-target inhibitors development has also been slow and none AQP-associated drugs has been applied for clinical anticancer therapy by far.

## Abbreviations

AQPs: Aquaporins
ADC: Adenocarcinoma
AVP: Vasopressin
BAC: Bronchoalveolar carcinoma
BBB: Blood-brain barrier
CCs: Cholangiocarcinomas
CML: Chronic myelogenous leukemia
CNS: Central nervous system
EGFR: Epidermal growth factor receptor
ERE: Estrogen-response element
ERK: Extracellular receptor kinase
FGF-2: Fibroblast growth factor-2
HCCs: Hepatocellular carcinomas
MCCs: Metastatic colorectal carcinomas
NMO: Neuromyelitis optica
NPA: Asparagine-proline-alanine
NSCLC: Non-small cell carcinoma
OAPs: Orthogonal arrays of particles
PKA: cAMP-protein kinase
VEGF: Vascular endothelial growth factor
5′-DFUR: 5′-deoxy-5-fluoropyrimidine nucleosides
